# Enhancing Balance Control in Aging Through Cerebellar Theta-Burst Stimulation

**DOI:** 10.1007/s12311-025-01915-x

**Published:** 2025-10-08

**Authors:** Ashwini Sansare, Madison Weinrich, Jessica A. Bernard, Yuming Lei

**Affiliations:** 1 Department of Kinesiology and Sports Management, Texas A&M University, College Station, TX USA; 2 Department of Psychological and Brain Sciences, Texas A&M University, College Station, TX USA; 3 Texas A&M Institute for Neuroscience, Texas A&M University, College Station, TX USA

**Keywords:** Standing Balance, Postural Sway, Transcranial Magnetic Stimulation (TMS), Neuromodulation, Cerebellar Brain Inhibition (CBI)

## Abstract

The cerebellum undergoes significant age-related changes linked to poor balance in older adults. Although multi-session cerebellar iTBS combined with rehabilitation has been used in some clinical populations, its isolated effects in community-dwelling healthy older adults remain unknown, particularly in context of balance control and underlying cerebellar-motor cortex (M1) interactions. We tested whether a single-session, sham-controlled, cerebellar iTBS-only intervention could modulate balance and cerebellar-motor cortex (M1) interactions in community-dwelling older adults without neurological disease. The effects of cerebellar intermittent theta-burst stimulation (iTBS) on balance control and underlying cerebellar-motor cortex (M1) interactions in this population remain unclear. We investigated whether cerebellar iTBS led to [1] improved standing balance, and [2] changes in cerebellar-M1interactions measured using cerebellar brain inhibition (CBI) in older adults. Forty older adults were randomized to receive Active (*n* = 20) or Sham (*n* = 20) iTBS to the right lateral cerebellum. We measured postural sway (95% ellipse area of the center of pressure) during standing and CBI before iTBS and at multiple time points up to 30 min post-stimulation. Compared to sham, a single session of active iTBS reduced postural sway, with balance improvements sustained for at least 30 min post-stimulation. Cerebellar iTBS did not significantly alter CBI. Our results support the neuroplastic potential of the cerebellum as a viable target for therapeutic interventions aimed at improving balance in aging, potentially influencing circuits beyond direct cerebellar-M1 motor pathways.

**Supplementary Information:**

The online version contains supplementary material available at 10.1007/s12311-025-01915-x.

## Introduction

Adapting rapidly to changes in the environment is extremely critical for maintaining upright balance. Adaptation is primarily driven by the cerebellum, which generates internal predictions about sensory consequences of motor commands and refines motor commands when sensory prediction errors arise [1–3]. The cerebellum is impacted by aging, which in turn could negatively impact these cerebellar functions. In advanced age, there is a reduction in cerebellar volume, in both white and gray matter [4–7]. Further, the cerebellum communicates with the primary motor cortex (M1) through closed-loop circuits via the thalamus [8–12]. Cerebellar-M1 networks show widespread degradation in older adults compared to young adults [13–15]. Overall, alterations in cerebellar structure and functional networks have been implicated in age-related declines in motor performance [7, 16, 17], cognition, and balance [5]. Further, recent work on resting-state functional connectivity between the cerebellum and M1 has demonstrated a direct link between age-related differences in cerebellar-M1 connectivity and postural sway during standing [18]. Overall, the strong relationship between motor and balance deficits, and age-related cerebellar structural and functional changes underscores the critical role the cerebellum plays in maintaining upright balance, particularly in advanced age.

While recent work on cerebellar neuromodulation has been shown to improve motor behavior, much of it has been focused on the use of transcranial direct current stimulation (tDCS). For example, cerebellar tDCS showed improved motor learning and locomotor adaptation in healthy young adults [19], and in initial skill acquisition during learning an upper limb motor sequence task [20] Recent work using cerebellar tDCS in conjunction with functional magnetic resonance imaging (fMRI) demonstrated alterations in resting state connectivity [21] as well as cortical changes in functional activation during a sequence learning task and a working memory task in young healthy adults [22]. With respect to postural control, cerebellar tDCS led to improved postural stability indices in older adults with high fall risk when combined with 2-week balance training intervention [23].

Another method of modulating the cerebellum involves using intermittent theta burst stimulation (iTBS), a form of repetitive transcranial magnetic stimulation (TMS) that produces long term potentiation effects but with shorter stimulation times than traditional repetitive TMS protocols. Cerebellar iTBS modulated cerebellar network connectivity [24] and M1 intracortical circuits [25], and improved patient-reported balance outcomes in stroke rehabilitation when combined with physical therapy [26, 27]. However, most prior studies have focused on clinical populations, such as individuals with stroke [26, 27], ataxia [28, 29], and multiple sclerosis [30] using multi-session protocols combined with rehabilitation, leaving it unclear whether a single session of cerebellar iTBS alone can modulate balance in otherwise healthy older adults. Moreover, these studies have either not tested balance related outcomes or have relied on clinical or patient-reported outcomes rather than objective force-plate measures of postural sway, and the short-term temporal profile of iTBS effects on balance in aging has not been characterized. Thus, the impact of cerebellar iTBS on objective postural sway measures in community-dwelling healthy older adults, a population particularly vulnerable to balance deficits and falls [31–33], remains largely unexplored. Discovery and development of stimulation techniques that can efficiently and effectively modulate balance, stand to have a positive impact on function and rehabilitation protocols in advanced age.

Furthermore, the impact of cerebellar neuromodulation on neurophysiological outcomes such as cerebellar-M1 interactions, which is one of the potential mechanisms hypothesized to drive the changes in balance and motor behavior, is unknown. Understanding the neurophysiological underpinnings is critical for advancing our mechanistic knowledge of cerebellar neuromodulation. Specifically, it can clarify how and why iTBS applied to the cerebellum produces changes in behavior. Such insights are essential for linking neural mechanisms to observed motor outcomes. Cerebellar-M1 interactions can be measured using cerebellar brain inhibition (CBI), a measure of the inhibitory control that cerebellar Purkinje cells typically exert over the M1 [34]. Delivering a conditioning TMS pulse to the cerebellum is thought to activate the Purkinje cells, leading to a reduction in corticospinal excitability, which can be quantified via motor-evoked potentials (MEP). Thus, a reduction in MEP amplitude from a dual stimulus (conditioning stimulus to cerebellum prior to test stimulus to M1) compared to a test stimulus to M1 alone, is referred to as cerebellar brain inhibition (CBI) and is a measure of the cerebellar output to M1. While CBI has been shown to be altered in older compared to young adults [35, 36], the effects of cerebellar neuromodulation on CBI are not yet known.

Here, we addressed these gaps by testing whether a single session of cerebellar iTBS improves balance and modulates cerebellar-M1 interactions in community-dwelling older adults without neurological disease. This design is distinct from prior work in that it [1] targets a healthy aging population [2], uses a single-session, sham-controlled, iTBS-only intervention, and [3] employs objective force-plate sway tracked over a continuous 30-min time course. In addition, this study is the first to include CBI as a mechanistic readout of cerebellar output to M1 in older adults under iTBS. We investigated the effects of iTBS to the right posterior lateral cerebellum on postural sway and on CBI in older adults. We hypothesized that cerebellar iTBS would lead to [1] reduced postural sway, indicating improved balance control, and [2] greater CBI, implying greater cerebellar output to M1 and in turn, greater cerebellar-M1 interactions.

## Methods

### Participants

**Fig. 1:**
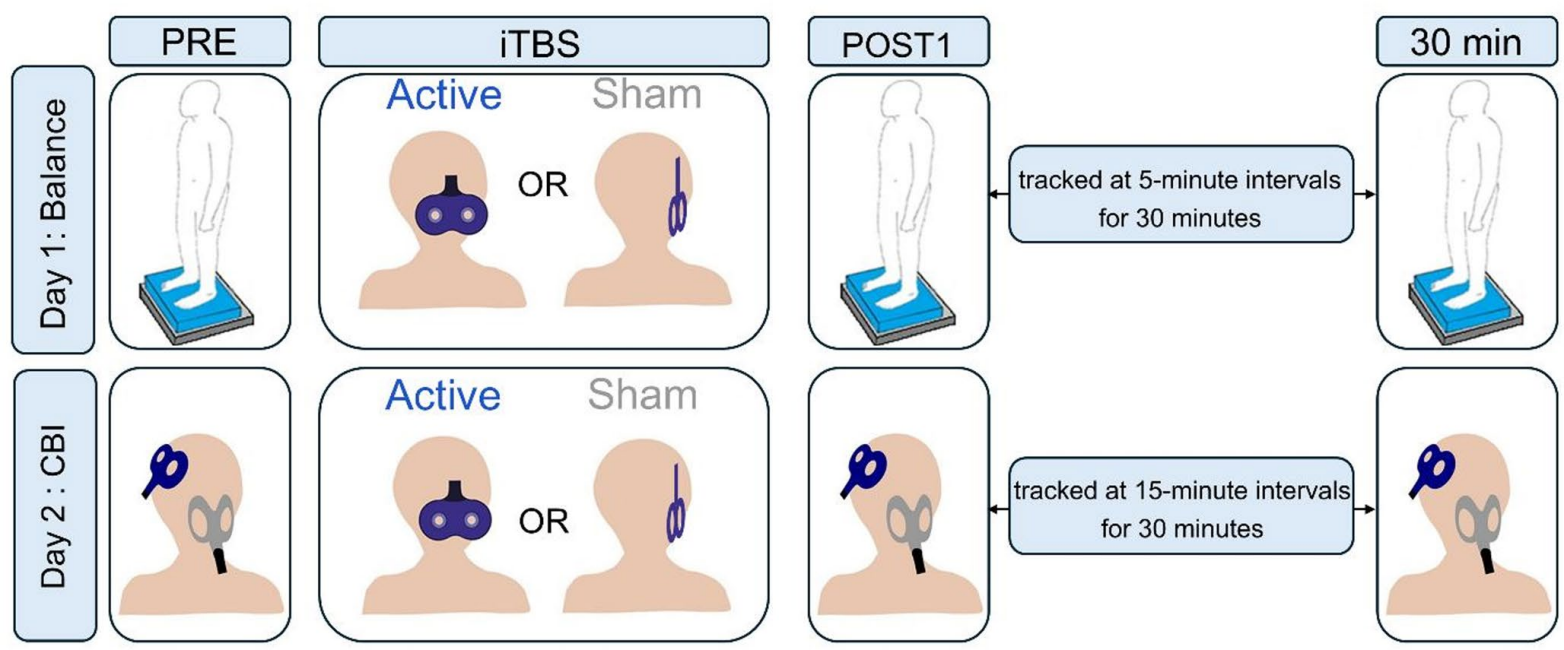
Experimental protocol

Forty older adults between 60 and 85 years of age (23 females) with no history of medical conditions or surgeries that might affect balance (e.g. vestibular disorders, knee or hip joint replacement within the past 2 years) were enrolled in this study. Other exclusion criteria include: (1) any factor that is a contraindication for the TMS stimulation [37]; (2) a diagnosis of a neurological or psychiatric disorder; (3) a score below 18 on the Montreal Cognitive Assessment (MOCA) [38]. All individuals self-reported being right-handed. Data was collected in a two-visit study, with the effects of iTBS on balance and CBI investigated on the first and second visit, respectively. The two visits were separated by a 7–10-day washout period. Participants were randomly assigned to either the active cerebellar iTBS group (*n* = 20) or the sham group (*n* = 20) using a computer-generated MATLAB random sequence. Group assignment was known only to the operator administering the iTBS to deliver the appropriate stimulation and was not revealed to the testers conducting the postural sway and CBI assessments. Although testers may have been able to infer group assignment by observing coil orientation in the sham condition, the outcome measures were entirely objective (i.e., postural sway recorded via force plate software and CBI measured via EMG and signal processing software) and thus unlikely to be influenced by assessor bias. All participants were blinded to group assignment. Figure 1 shows the experimental protocol. We chose a between-subject design over a within-subject crossover design for two main reasons. First, we did so to avoid potential carryover effects of cerebellar stimulation in the same group of older adults. Second, we used two groups to reduce participant burden and minimize attrition. Our protocol already required two visits—one for assessing changes in balance and another for neurophysiological measurements. A within-subject design would have required two additional visits for sham stimulation assessments, extending the study to four visits over a 4–6 week period (with 7–10 days between visits). All procedures were approved by the Texas A&M University Institutional Review Board, and all participants provided informed written consent in accordance with the Declaration of Helsinki (Clinical trial number: not applicable).

### Intermittent theta burst stimulation (iTBS)

A Magstim Rapid2 system (Magstim, UK) with a 70 mm figure of eight coil was used for administering iTBS. We elected to use a figure-of-eight coil for iTBS due to its safety profile in high-frequency protocols. While double-cone coils are often used for cerebellar brain inhibition (CBI), they exhibit a slightly higher seizure incidence (~ 0.12 vs. ~0.08 per 1,000 sessions) across all TMS paradigms [39]. Notably, no studies have systematically evaluated seizure risk or adverse events when delivering iTBS with a double-cone coil—whether over the cerebellum or other brain regions—leaving safety in this context unquantified. In contrast, the figure-of-eight coil remains most widely used for iTBS, with well-established tolerability, defined safety parameters, and regulatory acceptance.

We determined participant’s resting motor threshold (RMT), as the lowest intensity of stimulation that elicited a motor-evoked potential (MEP) of at least 50 µV in the relaxed right first dorsal interosseus muscle in a minimum of 5 of 10 trials. Surface electromyographic (EMG) was recorded using disposable electrodes (Ag-AgCl; 10 mm diameter). EMG signals were amplified and filtered (bandwidth 30–2000 Hz) with a Neurolog System amplifier (Digitimer, UK) and then converted to digital data with a sampling rate of 10 kHz with an A/D converter (CED Micro 1401, Cambridge Electronic Design, UK). iTBS was delivered at 75% of RMT, wherein bursts of high-frequency stimulation (three biphasic waveform pulses at 50 Hz) were applied at 5 Hz, every 10 s for a total of 600 pulses [40]. The coil was placed over the right lateral cerebellar hemisphere, 3 cm lateral to the midline and 1 cm below the inion of the occipital bone, with the handle pointed superiorly. We chose the right cerebellum because the right cerebellum projects to the contralateral motor cortex. We wanted to target the dominant motor cortex in our cohort of right-handed individuals. For the sham group, iTBS was performed using the same parameters at the same location but with the coil held perpendicular to the scalp over the cerebellum. This replicated the clicking sound of iTBS and the side of the coil made contact with the skull but the participant did not receive any stimulation. The above iTBS protocol was administered twice—once during the balance task and once during the CBI measurement—depending on group assignment (active or sham).

### Balance Measurement

Postural sway was assessed using an Advanced Mechanical Technology Incorporated Accusway force platform (Watertown, MA). Participants were asked to stand as still as possible with their hands crossed across their chest and their feet close together for one minute under four different conditions presented in random order: eyes open on firm surface (EO), eyes closed on firm surface (EC), eyes open on foam surface (EOF), eyes closed on foam surface (ECF). Given the large variability of functional abilities even in healthy older adults, evaluating balance under the EO condition may not be challenging enough (ceiling effect) while ECF condition might be too difficult (floor effect). To address both ceiling and floor effects, we selected the condition that produced the greatest sway from these four as the most challenging one and another trial of this condition was repeated for a baseline measure (PRE). For all participants in our study, the most challenging condition that was also completed independently without requiring the participant to step off or hold on for support was ECF. This condition was then repeated immediately after receiving iTBS (POST1) and at 5-minute intervals (POST2, POST3, POST4, POST5, POST6) until thirty minutes elapsed following iTBS stimulation. To optimize the ability to detect behavioral changes at regular intervals while minimizing fatigue, only the ECF condition was collected during the follow-up period. The center of pressure (COP) was recorded for one minute with a sample rate of 50 Hz. To isolate the low-frequency postural sway, we applied a 9th order Butterworth filter with a 20 Hz cutoff frequency. The 95% ellipse area of COP i.e. the area of the ellipse that encompasses 95% of the measured COP, was calculated using previously established methods to quantify postural sway [41] and was the primary outcome measure of interest.

### Cerebellar Brain Inhibition (CBI)

To assess CBI, we adopted a paired pulse paradigm (DuoMAG-MP Dual, DEYMED, Czech Republic). A test stimulus (TS) was delivered using a 70 mm figure of eight coil over the left first dorsal interosseous (FDI) muscle hotspot that elicited an ~ 1 mV MEP in 5 consecutive trials. A conditioning stimulus (CS) was delivered to the cerebellum using a double-cone coil placed 3 cm lateral and 1 cm inferior to the inion at 70% of the maximum stimulator output. The TS over left M1 was preceded by a CS over the right cerebellum by an interstimulus interval (IS) of 5 ms. Because the double-cone coil has a wider field that may activate the corticospinal tract during cerebellar stimulation, some protocols have selected CS intensity such that it was 5–10% below the active motor threshold determined by delivering brainstem stimulation using a double cone coil centered over the inion. However, we found that during pilot testing, our older adult participants did not tolerate brainstem stimulation well and we did not elicit any MEPs for some participants. Thus, we chose to use a pre-determined intensity for CS like in several CBI studies [36, 42] that was tolerable and yet able to elicit CBI. To verify that we were not potentially stimulating corticospinal tracts, we delivered 3 pulses at CS and checked the resultant EMG activity in the FDI muscle. None of our participants showed any FDI muscle activation in response to cerebellar stimulation using CS intensity. Two sets of ten trials were delivered with a 2-minute break between the two sets. There was a total of twenty trials, ten with TS only and ten with TS preceded by CS, presented in a random order. CBI was calculated by expressing the size of the conditioned MEP as a percentage of the size of the test MEP [(conditioned MEP/test MEP) × 100]. Greater CBI implies greater output from CB to M1, and in turn, greater the CB-M1 interactions. An optically tracked neuronavigation system (Brainsight, Rouge Research Inc, Montreal, Canada) ensured accurate and repeatable coil placement throughout the experiment. We performed a CBI measure at baseline and immediately following cerebellar iTBS (POST1), at 15 min (POST2) and 30 min (POST3) following the cessation of iTBS.

### Statistical Analysis

We performed all statistical analyses in in SPSS (Released 2023. IBM SPSS Statistics for Windows, Version 29, Armonk, NY: IBM Corp). To evaluate the effects of iTBS on sway area, we analyzed post-intervention values relative to baseline. Because baseline sway area can vary across individuals, we analyzed change-from-baseline values, which directly account for individual variability at baseline and provide a robust test of group differences in post-intervention change. For each participant, sway area at each post-intervention timepoint (POST1–POST6) was expressed as a change from the baseline measurement (POST1 – PRE, POST2 – PRE, and so on). These change scores served as the dependent variable in a 2 (Group) × 6 (Time) mixed ANOVA. Significant effects were followed up with post hoc pairwise comparisons using Bonferroni correction. We assessed assumptions of homoscedasticity and normality, respectively, by Levene’s and Shapiro–Wilk tests in addition to visual examination. The assumption of sphericity was tested using Mauchly’s test, and if sphericity was violated, a Greenhouse-Geisser correction factor was applied.

Because our primary hypothesis focused on detecting changes in balance following iTBS, we conducted an a priori power analysis using G*Power (version 3.3; [43]) based on changes in the 95% confidence ellipse area of the COP. Assuming a significance level of α = 0.05 and power (1–β) = 0.80, the power analysis indicated that a total of 34 participants (17 per group: Active and Sham) would be sufficient to detect a medium effect size (f = 0.25). Previous studies assessing balance interventions in older adults have reported partial eta squared values ranging from 0.06 to 0.12 [44–47], corresponding to *f* values of 0.25–0.36. Therefore, our assumption of *f* = 0.25 is both conservative and consistent with prior observed effects on COP ellipse area. To account for potential attrition or missing data, we recruited 20 participants per group.

## Results

The demographic information, including the age, sex, race, MOCA scores and education levels, has been reported in Table 1. The descriptive statistics for 95% COP area and CBI are displayed in Table 2.

**Table 1:** Demographic information, including the mean, standard deviations and ranges where applicable, for age, sex, race, ethnicity, MOCA scores and education level

		Mean	Std Dev	Range
Age (years)	Active	70	6.5	60–84
	Sham	71	6.8	60–82
Sex	Active	12 females, 8 males		
	Sham	12 females, 8 males		
Height (m)	Active	1.68	0.89	1.55–1.90
	Sham	1.67	1.06	1.47–1.85
Weight (kg)	Active	74.7	17.4	48–111
	Sham	77.2	16.7	54–103
MOCA scores	Active	27	2	21–30
	Sham	28	2	25–30
Education (in years)	Active	17	3	12–19
	Sham	15	2	12–19
Test stimulus (TS) intensity for CBI (%MSO)	Active	63	20	29–95
	Sham	60	14	38–84

**Table 2:** Mean and standard deviations for 95%COP area and CBI split by group (Active/Sham)

		Active			Sham		
		n	Mean	Std Dev	n	Mean	Std Dev
95%COParea	PRE	20	46.49	17.64	20	40.82	16.94
	POST1	20	36.18	14.90	20	47.87	32.14
	POST2	20	34.85	12.06	20	44.40	25.21
	POST3	20	34.08	15.89	20	39.78	19.93
	POST4	20	34.02	15.84	20	41.14	30.06
	POST5	20	34.37	15.43	20	40.36	21.20
	POST6	20	32.55	13.20	20	41.45	23.45
CBI	PRE	18	91.86	26.55	17	88.23	15.47
	POST1	18	85.96	28.36	17	92.76	20.99
	POST2	18	84.24	22.71	17	95.54	25.09
	POST3	18	79.95	25.35	17	79.67	20.49

### Effect of iTBS on Balance

**Fig. 2:**
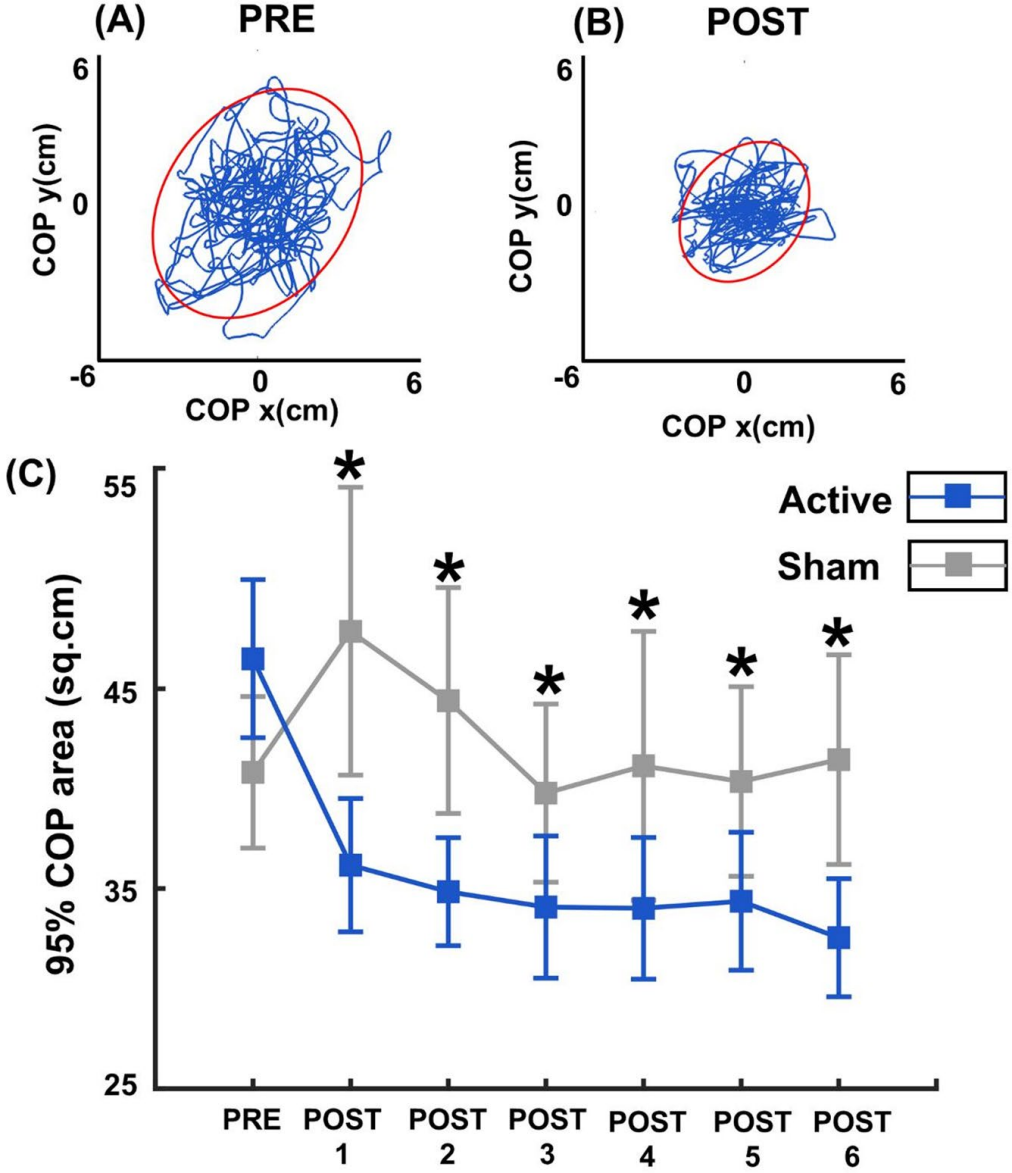
Panels **A** and **B **show 95% COP area before and after receiving cerebellar iTBS, respectively, in a representative participant in the Active group. Panel **C** shows the group averages for Active (blue) and Sham (grey) groups over 5 min intervals till 30 min following cessation of iTBS. Error bars indicate standard error of the mean. Asterisks indicate statistically significant reduction in the change in 95% COP area (*p* < 0.05) at post timepoints relative for Active compared to Sham group with Bonferroni corrected posthoc comparisons

Results from the 2 (group) x 6 (time) ANOVA revealed a significant main effect of Group (F = 9.600, *p* = 0.004, η2 = 0.202), indicating that the Active group demonstrated greater reductions in sway relative to baseline compared with the Sham group (Fig. 2). Box and whisker plots combined with line plots illustrating individual trajectories of 95% COP area over time have been included in Supplementary Fig. 1. Pairwise post hoc comparisons using Bonferroni corrections revealed that the Active group showed significantly greater reductions in sway area than the Sham group at each of the six post-intervention timepoints (*p* = 0.006, *p* = 0.002, *p* = 0.018, *p* = 0.040, *p* = 0.011, *p* = 0.003 for POST1 to POST6, respectively). There was no significant main effect for time (F = 2.326, *p* = 0.065, η2 = 0.058). Additionally, group × time interaction was not significant (F = 0.734, *p* = 0.558, η2 = 0.019), suggesting that the magnitude of change in the sway for either group (i.e. the decrease in sway for the Active and increase in sway for the Sham) was consistent across the post-intervention timepoints, implying that learning or fatigue did not progressively affect either group across the 30 min period.

#### Effect of iTBS on CBI

**Fig. 3:**
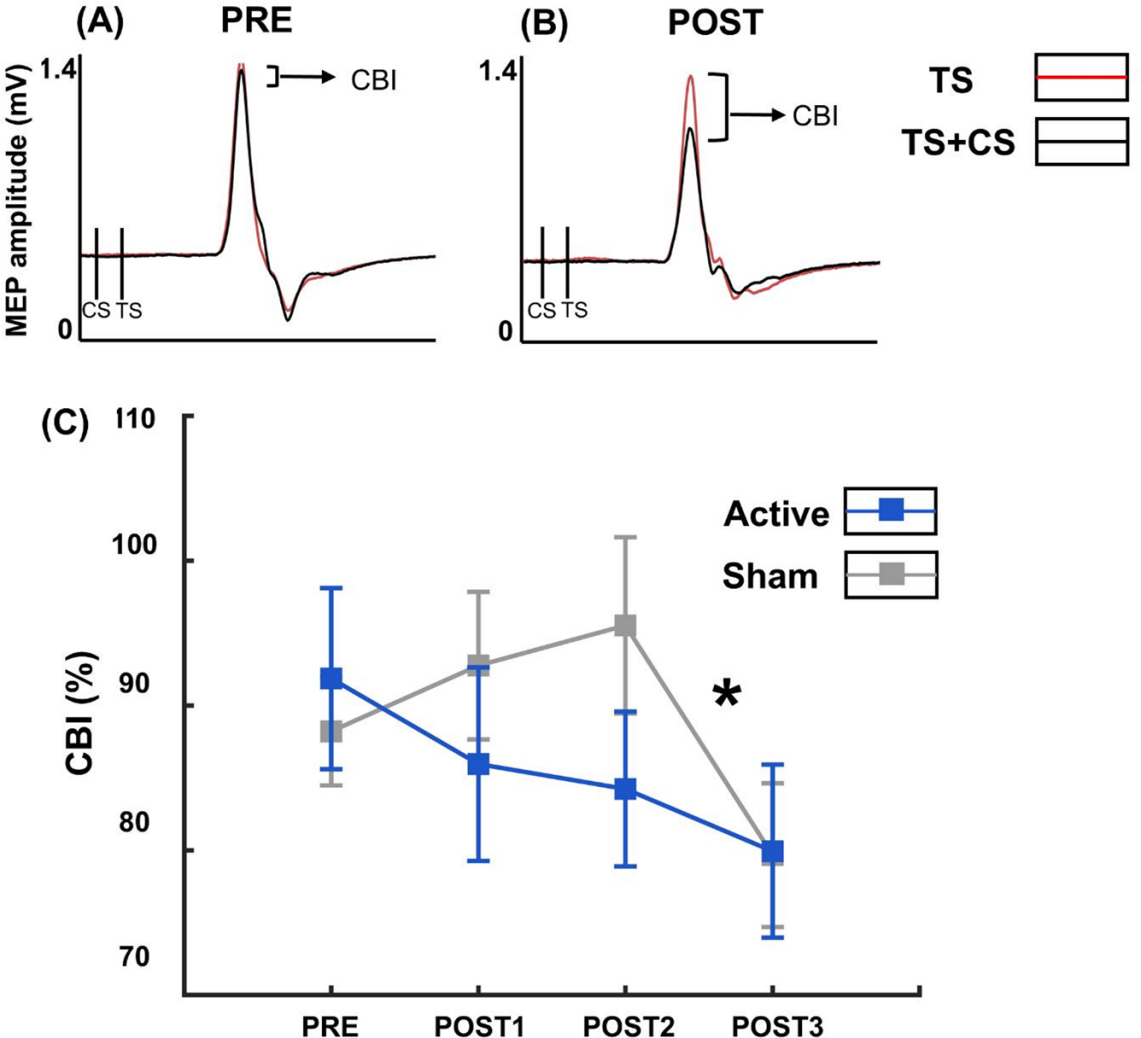
Panels **A** and **B** show CBI i.e. ratio of MEP after test stimulus (red) over MEP after a test stimulus preceded by a conditioning stimulus (black) to the cerebellum, before and after receiving cerebellar iTBS, respectively, in the same representative participant as Fig. 2 in the Active group. Panel **C** shows the group averages for Active (blue) and Sham (grey) groups over 5 min intervals till 30 min following cessation of iTBS. Error bars indicate standard error of the mean. Asterisk indicates that irrespective of group assignment, there is a statistically significant reduction in CBI at POST 3 vs. POST2 with Bonferroni corrected posthoc comparisons

Two participants from the Active and three from the Sham group did not complete this visit due to not being able to tolerate the CS over the cerebellum. Results from 2 (group) x 3 (time) ANOVA showed that there was no significant effect for group (F = 1.570, *p* = 0.219, η2 = 0.045) nor a group by time interaction (F = 0.962, *p* = 0.387, η2 = 0.028) (Fig. 3). Overall, we saw numerical reductions in CBI in the Active group, but nothing was statistically significant on a group level between Active and Sham. However, there was a significant main effect for time (F = 3.633, *p* = 0.032, η2 = 0.099), indicating that irrespective of group assignment, CBI changed over time. Pairwise post hoc comparisons using Bonferroni corrections revealed that the change in CBI at POST3 was significantly lower than that at POST2 (*p* = 0.037). Box and whisker plots illustrating individual trajectories of CBI over time have been included in Supplementary Fig. 1.

## Discussion

This sham-controlled study examined the immediate and short-term (30-minute) effects of cerebellar intermittent theta burst stimulation (iTBS) on postural sway and cerebellar-M1 interactions in older adults. iTBS significantly reduced postural sway, demonstrating improved balance control that was sustained for at 30 min post-stimulation. However, there were no significant effects of iTBS on CBI. This is one of the first studies to not only investigate the effect of cerebellar iTBS on objective measures of balance in older adults but also investigate the effect on cerebellar-M1 neurophysiology.

### Improved Standing Balance Control Following Cerebellar Neuromodulation

Cerebellar iTBS led to a significant reduction in postural sway and these improvements were retained for at least 30 min following iTBS cessation. While prior work on the effects of cerebellar iTBS on network connectivity showed evidence of changes in cerebello-cortical circuits following neuromodulation [24], our results offer direct evidence of resultant motor behavior changes.

While there was a reduction in the postural sway immediately following iTBS (POST1) in the active group, this reduction was not statistically significant. Conversely, the sham group exhibited a significant increase in postural sway at POST1, despite receiving no active cerebellar stimulation. The movement from sitting during iTBS to standing for the postural assessment may explain the patterns seen in both groups. This postural transition, particularly in older adults, may have led to greater sway at the first POST measurement in the sham group and may have undermined the potential beneficial effects of iTBS in the active group, thus leading to smaller than expected effect on postural sway.

We found that the greatest reduction in postural sway in the active group occurred around 10–20 min following iTBS, indicating that there may be a window of optimal cerebellar function following a single iTBS session, where the greatest improvement may be potentially noted. While fatigue may have affected both groups, particularly in the latter half of the protocol, a continued improvement in the Active group around 10–20 min that was retained at the 30-minute follow-up timepoint highlights the neuroplastic potential of cerebellar neuromodulation using iTBS. This temporal profile is consistent with prior work showing that a single session of iTBS (600 pulses) is reported to increase cortical excitability indexed by MEP amplitude for ~ 20–30 min following stimulation [40]. Our results are the first to not only track and report the effects of a single session of cerebellar iTBS on balance control across a short time interval, while also extending Huang et al.’s [40] findings to older adults.

Interestingly, the measurable reduction in postural sway observed in our older adult participants contrasts with the often subtle behavioral effects reported in younger adults following cerebellar stimulation. This difference may reflect a greater ‘behavioral leverage’ of cerebellar neuromodulation in aging, potentially due to age-related declines in cerebellar structure and function that leave more capacity for plastic change. In other words, older adults may have more room for improvement, allowing iTBS to produce more pronounced behavioral effects. These findings suggest that targeting cerebellar circuits in aging populations may offer enhanced translational potential for interventions aimed at improving balance and reducing fall risk.

### No Significant Improvement in Cerebellar-M1 Interactions Following Cerebellar Neuromodulation

We did not see any statistically significant changes in CBI ratio after iTBS. Our results showed that CBI ratio numerically decreased immediately following active cerebellar iTBS, but none of these changes reached statistical significance. The lack of significant findings could be due to the high variability in the CBI response seen following iTBS, even in the sham group. Of the three studies to date investigating CBI in older adults, Rurak et al. [35] reported a reduced CBI, Mooney et al. [36] showed an increase in CBI in older adults, while Van Malderen et al. [48] reported no differences in CBI between young and older adults at rest. Further, while CBI has shown moderate reliability in younger adults [49], it showed poor reliability in older adults [35]. The mixed nature of the CBI findings in these studies along with our results supports the high degree of variability seen in the aging population with respect to the cerebellar-M1 interactions. Consistent with these prior reports, our findings suggest that CBI may be less reliably modulated in older adults, reflecting an age-dependent dissociation in cerebellar plasticity. Thus, the postural improvements we observed may not be mediated solely by cerebellar-M1 interactions but rather through compensatory or alternative pathways, such as cerebellar-prefrontal or more distributed motor-cognitive networks. This perspective highlights the importance of considering broader network contributions when interpreting cerebellar stimulation effects in older adults and suggests that behavioral gains may emerge through multiple, potentially compensatory mechanisms. Despite its reported low reliability and variability of response in older adults, we selected CBI as an outcome measure for our study for two primary reasons. First, at the time of this study’s development, only two prior studies had examined CBI in older adults [35, 36], and as such, it remained unclear whether the observed inconsistencies were due to inherent reliability issues with the measure itself or to the lack of consensus regarding stimulation and recording protocols. Second, given our specific interest in assessing immediate neurophysiological interactions between the cerebellum and primary motor cortex (M1), CBI was the most direct and theoretically appropriate measure available. Our findings contribute to a limited body of research examining cerebellar-M1 connectivity in older adults and underscore the need for improved standardization of CBI protocols. Importantly, they highlight the complexity and variability of cerebellar modulation in aging—an essential consideration for future studies aiming to use cerebellar stimulation to enhance motor function in this population.

It is also possible that certain methodological choices contributed to the high variability observed in the CBI response to iTBS. We have listed the key stimulation parameters used in the present study vs. those used in prior work on CBI in older adults in Table 3 below for ease of comparison.

**Table 3:** Comparison of key stimulation parameters against prior work on CBI in older adults

Study	TS intensity	CS intensity	Number of pulses	Timing of ISI	Session spacing	Results summary
Rurak et al., 2022	90% RMT	5% below brainstem AMT	8 TS only, 8 TS + CS at 3 ms, 8 TS + CS at 5 ms in one block, pseudo-randomized with intertrial interval of 5 ms. Four blocks (2 with figure of eight coil, two with double cone coil).	3ms and 5 ms	Two 2-hr sessions, 7 days apart	CBI was lesser in older compared to younger adults, Intraclass correlation coefficients showed poor stability of CBI as a measure in older adults.
Mooney et al., 2022	Intensity that elicits an ~ 1mV MEP in FDI	5% below brainstem AMT, if no MEP evoked, then 75%MSO	10 trials for each condition in older adults (30 total presented in random order)	5ms and 7 ms	Single session	CBI was greater in older compared to younger adults.
Van Maldren et al., 2025	Intensity that elicits an ~ 1mV MEP in FDI	65% of MSO	20 TS only, 20 TS + CS presented in random order for resting CBI, two blocks of task related CBI with two sets of 10 TS only and 10 TS + CS trials in each block.	5 ms	Single session	No age-related difference in CBI at rest or during anticipation of bimanual task.
Present study	Intensity that elicits an ~ 1mV MEP in FDI	70% of MSO	Two sets of 10 TS only, 10 TS + CS at each timepoint. Four timepoints measured in ~ 45 min (baseline, following iTBS immediately i.e. at 0-min, 15-min and 30- min POST iTBS).	5 ms	Single session	Cerebellar iTBS significantly reduced postural sway in older adults but did not alter CBI significantly.
TS = Test Stimulus, CS = Conditioning stimulus, AMT = Active Motor Threshold, ISI = Interstimulus Interval, MSO = Maximum stimulator output						

In particular, we employed a fixed CS intensity in our protocol given the tolerability concerns in older adults seen in our pilot testing and supported by previous work on CBI [42, 48, 50]. While this approach prioritized participant comfort, it inevitably limited the ability to individualize stimulation parameters. As a result, variability in individual cortical excitability and cerebellar responsiveness may have reduced the effectiveness and consistency of cerebellar stimulation across participants. This trade-off highlights the ongoing challenge of balancing methodological rigor with participant-specific constraints in neurostimulation studies involving older adults. Additionally, our neurophysiological measures were indexed via the FDI hand muscle, which is not directly involved in the postural control outcomes we measured. This approach, however, was methodologically necessary and is consistent with the established protocols listed in Table 3. Nonetheless, this approach enabled us to use a well-established and tolerable protocol while still providing a physiologically grounded measure of cerebellar–motor cortical interactions.

Several studies have used CBI as a marker for investigating whether the cerebellar inhibition on M1 can be modulated i.e. increased or decreased, following behavioral tasks such as motor sequencing [51, 52], locomotor [53]and upper limb adaptation [54] tasks or during motor preparation of simple reaction time tasks [55], where CBI was generally reduced during early learning. However, this is the first study to use CBI as a marker for changes following direct cerebellar modulation using non-invasive brain stimulation. The CBI measures were taken immediately before and after the iTBS session, and at the 15 min- and 30-minute timepoint following iTBS. The significant main effect of time suggests that repeated cerebellar stimulation itself may influence CBI, independent of group assignment. This repeated stimulation of the cerebellum, both with paired-pulse stimulation to measure CBI at timed intervals and with theta burst stimulation to modulate cerebellar activity, all within a span of 45 min, may have led to the mixed CBI results with high variability that was seen in our cohort. Thus, CBI may not be a stable measure for *repeated* measurements of cerebellar-M1 interactions before and after a non-invasive brain stimulation protocol, particularly in older adults.

Lastly, the cerebellum is known to play a role in both postural control [56–58] as well as cognitive functions [59–64] Specifically, cerebellar lobules V, VI, and VIII have well established connections with the motor cortex, highlighting its role in motor control while the Crus I and II are integrated into cognitive networks via their connections to the prefrontal and parietal cortex [65–68]. Given the anatomical structure of the cerebellum, non-invasive stimulation targeting the deeper motor regions (such as Lobules V, VI) likely also affects the cognitive regions (Crus I and II). Thus, in studies employing cerebellar stimulation - including the present work – it is probable that both motor and cognitive networks are modulated [69]. While CBI measures the cerebellar-motor cortical interactions, the coil placement over the posterior cerebellum may also stimulate Crus I and II, which are linked to prefrontal regions. This raises the possibility that observed effects may be mediated through cerebellar-thalamic-prefrontal pathways rather than solely through cerebellar-M1 circuits. Supporting this idea, recent evidence has shown that cognition moderates the relationship between sensorimotor connectivity and balance in older adults—those with lower cognitive performance exhibited a stronger association between connectivity and balance outcomes [18]. These findings suggest that cognitive networks may play a compensatory or modulatory role in balance control and highlight the need to consider the broader, integrative role of the cerebellum when interpreting the effects of cerebellar stimulation in aging populations. Future research incorporating neuroimaging to track changes in cerebellar-thalamo-cognitive networks, along with comprehensive assessments of cognitive domains such as working memory and verbal fluency, would enhance both the mechanistic understanding and translational relevance of these findings.

While our findings suggest a role for cerebellar mechanisms in age-related balance impairments, these changes are unlikely to be attributable to cerebellar dysfunction alone. Sensory dysfunction, which is more prevalent in older compared to younger adults, likely also contributes to impaired postural control. We did not directly assess sensory processing in the present study; however, future work incorporating measures such as somatosensory evoked potentials (SEPs) to compare younger and older adults would provide valuable insight into the relative contributions of cerebellar and sensory pathways to balance regulation. Also, while it is true that the cerebellar vermis plays a central role in posture and balance, we selected the lateral cerebellum for three key reasons: first, stimulation over lobules V–VI and VIII targets regions critical for motor control [62]; second, the lateral cerebellum projects to the primary motor cortex, enabling direct physiological assessment via CBI [70–72]; and third, our recent work demonstrated that postural sway during eyes-closed standing is significantly related to connectivity between the lateral cerebellum and M1 [18]. Nonetheless, future studies may benefit from directly comparing bilateral or midline-focused stimulation with lateral cerebellar stimulation to determine which approach most effectively targets axial postural control mechanisms.

### Limitations

While this work provides valuable insight into the neuroplastic potential of cerebellar neuromodulation, it is not without limitations. First, we only followed participants following a single session of iTBS for up to 30 min only. Future work should therefore investigate approaches to extend this window, including multi-session stimulation to leverage cumulative plasticity, dose-finding studies that systematically evaluate stimulation parameters (e.g., intensity, frequency, number of sessions), or pairing cerebellar iTBS with task-specific balance and gait training to promote longer-lasting functional gains. Establishing clear milestones, such as the minimal number of sessions required to induce lasting motor and balance improvements, durability of effects over weeks or months, and safety/tolerability thresholds, will be essential for advancing cerebellar iTBS toward clinical application. Such a roadmap would provide the necessary foundation before translating cerebellar iTBS into fall-prevention programs for older adults at risk. Although this time frame was based on prior work [40] demonstrating behavioral changes lasting up to 30 min post-stimulation, it is possible that effects on postural sway extended beyond this period. As such, our conclusions are limited to the immediate effects observed, and we cannot infer the presence or absence of sustained effects beyond this window, which poses a challenge for clinical translation where durable improvements are essential. Second, although our sample size was determined through a priori power analysis that accounted for anticipated attrition, the high variability observed in both outcome measures—particularly in the CBI—suggests that future studies with larger samples of older adults may provide greater statistical power and more stable estimates. Further, this would allow for exploratory investigation into factors that influence responsiveness to cerebellar iTBS and positive behavioral outcomes. Given our current sample size, formal responder analyses or correlations between sway and CBI changes were not feasible. We suggest that future studies with larger samples pursue these analyses to better understand individual variability and potential predictors of response. Third, the use of a fixed CS intensity, while necessary for tolerability in older adults, may have contributed to variability in CBI responses due to a lack of individualized stimulation based on cortical and cerebellar excitability. Next, the penetration depth of a figure-of-eight coil is limited, which poses challenges in selectively reaching deeper cerebellar structures. It is therefore possible that stimulation in the present study primarily engaged more superficial portions of lobule VIII, which contributes to motor control, and lobule VII, which has been implicated in cognitive functions that may indirectly affect balance [18]. Prior modelling and empirical work suggests that rTMS and theta-burst stimulation over the lateral cerebellum with a figure-of-eight coil can modulate Purkinje cell activity within these lobules and influence cerebello-thalamo-cortical interactions as measured by CBI [25, 73, 74] and have also been associated with changes in balance outcomes [26]. Nonetheless, the absence of individualized field modelling weakens the anatomical specificity of our approach. Future studies should incorporate computational modelling or leverage alternative technologies, such as transcranial focused ultrasound, which provide greater spatial precision and allow for deeper targeting of lobules V–VI without compromising focality. Finally, the sham procedure used in this study, coil placement perpendicular to the scalp, was selected to minimize the possibility of inadvertent brain stimulation seen with other sham protocols [75]. However, this approach does not fully reproduce the scalp sensations of active stimulation and may reduce the effectiveness of participant blinding, particularly in participants that are not TMS naive. Moreover, we did not assess whether participants could correctly guess their treatment arm, so the success of blinding remains uncertain. This limitation warrants caution in the interpretation of our findings, and future studies should consider alternative sham protocols, such as specialized sham coils, that more closely mimic active stimulation without inducing neural effects.

## Conclusion

In conclusion, our results suggest that cerebellar iTBS reduces postural sway, with balance improvements sustained for at least 30 min post-stimulation. Cerebellar iTBS did not alter CBI significantly. These findings point towards circuits beyond cerebellar-M1, such as cognitive circuits, that may be impacted by cerebellar neuromodulation. However, given the limitations of our study, future replication is needed to confirm our findings. Overall, these results support the neuroplastic potential of the cerebellum for restoring balance function in older adults and identify the cerebellum as a potential target for therapeutic interventions for inducing improvements in balance control.

## Supplementary Information

Below is the link to the electronic supplementary material.

Supplementary Material 1 (DOCX 463 KB)

## Data Availability

The data that support the findings of this study are available from the corresponding author upon reasonable request.

## References

[1] Wolpert DM, Miall RC, Kawato M. Internal models in the cerebellum. Trends Cogn Sci. 1998;2(9):338–47. 10.1016/S1364-6613(98)01221-2. 21227230 10.1016/s1364-6613(98)01221-2

[2] Wolpert DM, Ghahramani Z, Flanagan JR. Perspectives and problems in motor learning. Trends Cogn Sci [Internet]. 2001 Nov 1 [cited 2025 Jan 17];5(11):487–94. Available from: http://www.cell.com/article/S1364661300017733/fulltext 10.1016/s1364-6613(00)01773-3 11684481

[3] Bastian AJ. Learning to predict the future: the cerebellum adapts feedforward movement control. Curr Opin Neurobiol. 2006;16(6):645–9. 17071073 10.1016/j.conb.2006.08.016

[4] Raz N, Lindenberger U, Rodrigue KM, Kennedy KM, Head D, Williamson A, et al. Regional brain changes in aging healthy adults: general trends, individual differences and modifiers. Cereb Cortex. 2005;15(11):1676–89. 10.1093/cercor/bhi044. 15703252 10.1093/cercor/bhi044

[5] Bernard JA, Seidler RD. Relationships Between Regional Cerebellar Volume and Sensorimotor and Cognitive Function in Young and Older Adults. The Cerebellum. 2013;12(5):721–37. 23625382 10.1007/s12311-013-0481-z PMC3820158

[6] Han S, An Y, Carass A, Prince JL, Resnick SM. Longitudinal analysis of regional cerebellum volumes during normal aging. Neuroimage. 2020;220:117062. 32592850 10.1016/j.neuroimage.2020.117062 PMC10683793

[7] Hicks TH, Magalhães TNC, Jackson TB, Ballard HK, Herrejon IA, Bernard JA. Functional and structural cerebellar-behavior relationships in aging. Brain Struct Funct. 2025;230(1):1–24. 10.1007/s00429-024-02862-9. 10.1007/s00429-024-02862-9 PMC11926889 39692877

[8] Dum RP, Strick PL. An unfolded map of the cerebellar dentate nucleus and its projections to the cerebral cortex. J Neurophysiol. 2003;89(1):634–9. 10.1152/jn.00626.2002. 12522208 10.1152/jn.00626.2002

[9] Kelly RM, Strick PL. Cerebellar Loops with Motor Cortex and Prefrontal Cortex of a Nonhuman Primate. The Journal of Neuroscience [Internet]. 2003;23(23):8432. Available from: http://www.jneurosci.org/content/23/23/8432.abstract 10.1523/JNEUROSCI.23-23-08432.2003 PMC6740694 12968006

[10] Salmi J, Pallesen KJ, Neuvonen T, Brattico E, Korvenoja A, Salonen O, et al. Cognitive and motor loops of the human cerebro-cerebellar system. J Cogn Neurosci. 2010;22(11):2663–76. 10.1162/jocn.2009.21382. 19925191 10.1162/jocn.2009.21382

[11] Stoodley CJ, Valera EM, Schmahmann JD. Functional topography of the cerebellum for motor and cognitive tasks: an fMRI study. Neuroimage. 2012;59(2):1560–70. 21907811 10.1016/j.neuroimage.2011.08.065 PMC3230671

[12] Bernard JA, Orr JM, Mittal VA. Differential motor and prefrontal cerebello-cortical network development: Evidence from multimodal neuroimaging. Neuroimage. 2016;124:591–601. 26391125 10.1016/j.neuroimage.2015.09.022 PMC4651741

[13] Bernard JA, Peltier SJ, Wiggins JL, Jaeggi SM, Buschkuehl M, Fling BW, et al. Disrupted cortico-cerebellar connectivity in older adults. Neuroimage. 2013;83:103–19. 23792980 10.1016/j.neuroimage.2013.06.042 PMC3815977

[14] Uwisengeyimana J, de Nguchu D, Wang BA, Zhang Y, Liu D, Qiu Y. Cognitive function and cerebellar morphometric changes relate to abnormal intra-cerebellar and cerebro-cerebellum functional connectivity in old adults. Exp Gerontol. 2020;140:111060. 32814097 10.1016/j.exger.2020.111060

[15] Bernard JA, Ballard HK, Jackson TB. Cerebellar dentate connectivity across adulthood: a large-scale resting state functional connectivity investigation. Cereb Cortex Commun. 2021;2(3):1–13. 10.1093/texcom/tgab050. 10.1093/texcom/tgab050 PMC8436571 34527949

[16] Koppelmans V, Hirsiger S, Mérillat S, Jäncke L, Seidler RD. Cerebellar gray and white matter volume and their relation with age and manual motor performance in healthy older adults. Hum Brain Mapp. 2015;36(6):2352–63. 10.1002/hbm.22775. 25704867 10.1002/hbm.22775 PMC6869104

[17] Seidler R, Erdeniz B, Koppelmans V, Hirsiger S, Mérillat S, Jäncke L. Associations between age, motor function, and resting state sensorimotor network connectivity in healthy older adults. Neuroimage [Internet]. 2015;108:47–59. Available from: https://www.sciencedirect.com/science/article/pii/S1053811914010155 10.1016/j.neuroimage.2014.12.023 25514517

[18] Sansare A, Magalhaes TNC, Bernard JA. Relationships of functional connectivity of motor cortex, primary somatosensory cortex, and cerebellum to balance performance in middle-aged and older adults. Neurobiol Aging [Internet]. 2025 Mar 1 [cited 2024 Dec 3];147:1–11. Available from: https://linkinghub.elsevier.com/retrieve/pii/S019745802400201X 10.1016/j.neurobiolaging.2024.11.009 PMC11973825 39637518

[19] Jayaram G, Tang B, Pallegadda R, Vasudevan EVL, Celnik P, Bastian A. Modulating locomotor adaptation with cerebellar stimulation. J Neurophysiol. 2012;107(11):2950–7. 10.1152/jn.00645.2011. 22378177 10.1152/jn.00645.2011 PMC3378372

[20] Ballard HK, Goen JRM, Maldonado T, Bernard JA. Effects of cerebellar transcranial direct current stimulation on the cognitive stage of sequence learning. J Neurophysiol. 2019;122(2):490–9. 10.1152/jn.00036.2019. 31166807 10.1152/jn.00036.2019

[21] Maldonado T, Jackson TB, Rezaee Z, Bernard JA. Time Dependent Effects of Cerebellar tDCS on Cerebello-cortical Connectivity Networks in Young Adults. The Cerebellum 2024 24:1 [Internet]. 2025 Jan 10 [cited 2025 Mar 26];24(1):1–16. Available from: https://link.springer.com/article/10.1007/s12311-024-01781-z10.1007/s12311-024-01781-z39794631

[22] Maldonado T, Jackson TB, Bernard JA. Anodal cerebellar stimulation increases cortical activation: evidence for cerebellar scaffolding of cortical processing. Hum Brain Mapp. 2023;44(4):1666–82. 10.1002/hbm.26166. 36468490 10.1002/hbm.26166 PMC9921230

[23] Yosephi MH, Ehsani F, Zoghi M, Jaberzadeh S. Multi-session anodal tDCS enhances the effects of postural training on balance and postural stability in older adults with high fall risk: Primary motor cortex versus cerebellar stimulation. Brain Stimul [Internet]. 2018;11(6):1239–50. Available from: https://www.sciencedirect.com/science/article/pii/S1935861X18302481 10.1016/j.brs.2018.07.044 30017699

[24] Halko MA, Farzan F, Eldaief MC, Schmahmann JD, Pascual-Leone A. Intermittent Theta-Burst Stimulation of the Lateral Cerebellum Increases Functional Connectivity of the Default Network. Journal of Neuroscience [Internet]. 2014 Sep 3 [cited 2025 Jan 30];34(36):12049–56. Available from: https://www.jneurosci.org/content/34/36/12049 10.1523/JNEUROSCI.1776-14.2014 PMC4152606 25186750

[25] Koch G, Mori F, Marconi B, Codecà C, Pecchioli C, Salerno S, et al. Changes in intracortical circuits of the human motor cortex following theta burst stimulation of the lateral cerebellum. Clin Neurophysiol. 2008;119(11):2559–69. 18824403 10.1016/j.clinph.2008.08.008

[26] Koch G, Bonnì S, Casula EP, Iosa M, Paolucci S, Pellicciari MC, et al. Effect of cerebellar stimulation on gait and balance recovery in patients with hemiparetic stroke: a randomized clinical trial. JAMA Neurol. 2019;76(2):170–8. 30476999 10.1001/jamaneurol.2018.3639 PMC6439971

[27] Liao LY, Zhu Y, Peng QY, Gao Q, Liu L, Wang QH, et al. Intermittent theta-burst stimulation for stroke: primary motor cortex versus cerebellar stimulation: a randomized sham-controlled trial. Stroke. 2024;55(1):156–65. 10.1161/STROKEAHA.123.044892. 38037225 10.1161/STROKEAHA.123.044892

[28] Shi Y, Zou G, Chen Z, Wan L, Peng L, Peng H, et al. Efficacy of cerebellar transcranial magnetic stimulation in spinocerebellar ataxia type 3: a randomized, single-blinded, controlled trial. J Neurol. 2023;270(11):5372–9. 10.1007/s00415-023-11848-2. 37433893 10.1007/s00415-023-11848-2

[29] Bonnì S, Ponzo V, Caltagirone C, Koch G. Cerebellar theta burst stimulation in stroke patients with ataxia. Funct Neurol [Internet]. 2014 [cited 2025 Aug 23];29(1):41. Available from: https://pmc.ncbi.nlm.nih.gov/articles/PMC4172246/PMC4172246 25014048

[30] Mori F, Ljoka C, Magni E, Codecà C, Kusayanagi H, Monteleone F, et al. Transcranial magnetic stimulation primes the effects of exercise therapy in multiple sclerosis. J Neurol. 2011;258(7):1281–7. 10.1007/s00415-011-5924-1. 21286740 10.1007/s00415-011-5924-1

[31] Wingert JR, Welder C, Foo P. Age-related hip proprioception declines: effects on postural sway and dynamic balance. Arch Phys Med Rehabil. 2014;95(2):253–61. 23994251 10.1016/j.apmr.2013.08.012

[32] Piirtola M, Era P. Force platform measurements as predictors of falls among older people – a review. Gerontology. 2006;52(1):1–16. 10.1159/000089820. 16439819 10.1159/000089820

[33] Ambrose AF, Paul G, Hausdorff JM. Risk factors for falls among older adults: a review of the literature. Maturitas. 2013;75(1):51–61. 23523272 10.1016/j.maturitas.2013.02.009

[34] Ugawa Y, Uesaka Y, Terao Y, Hanajima R, Kanazawa I. Magnetic stimulation over the cerebellum in humans. Ann Neurol. 1995;37(6):703–13. 10.1002/ana.410370603. 7778843 10.1002/ana.410370603

[35] Rurak BK, Rodrigues JP, Power BD, Drummond PD, Vallence AM. Reduced cerebellar brain inhibition measured using dual-site TMS in older than in younger adults. Cerebellum. 2022;21(1):23–38. 10.1007/s12311-021-01267-2. 33880658 10.1007/s12311-021-01267-2

[36] Mooney RA, Ni Z, Shirota Y, Chen R, Ugawa Y, Celnik PA. Age-related strengthening of cerebello-cortical motor circuits. Neurobiol Aging. 2022;118:9–12. 35810524 10.1016/j.neurobiolaging.2022.04.016 PMC11753508

[37] Rossi S, Hallett M, Rossini PM, Pascual-Leone A, Avanzini G, Bestmann S, et al. Safety, ethical considerations, and application guidelines for the use of transcranial magnetic stimulation in clinical practice and research. Clin Neurophysiol. 2009;120(12):2008–39. 19833552 10.1016/j.clinph.2009.08.016 PMC3260536

[38] Nasreddine ZS, Phillips NA, Bédirian V, Charbonneau S, Whitehead V, Collin I, et al. The Montreal cognitive assessment, MoCA: a brief screening tool for mild cognitive impairment. J Am Geriatr Soc. 2005;53(4):695–9. 10.1111/j.1532-5415.2005.53221.x. 15817019 10.1111/j.1532-5415.2005.53221.x

[39] Lerner AJ, Wassermann EM, Tamir DI. Seizures from transcranial magnetic stimulation 2012–2016: Results of a survey of active laboratories and clinics. Clin Neurophysiol [Internet]. 2019 Aug 1 [cited 2025 Jul 13];130(8):1409. Available from: https://pmc.ncbi.nlm.nih.gov/articles/PMC7274462/10.1016/j.clinph.2019.03.016 PMC7274462 31104898

[40] Huang YZ, Edwards MJ, Rounis E, Bhatia KP, Rothwell JC. Theta burst stimulation of the human motor cortex. Neuron. 2005;45(2):201–6. 15664172 10.1016/j.neuron.2004.12.033

[41] Prieto TE, Myklebust JB, Hoffmann RG, Lovett EG, Myklebust BM. Measures of postural steadiness: differences between healthy young and elderly adults. IEEE Trans Biomed Eng. 1996;43(9):956–66. 9214811 10.1109/10.532130

[42] Fernandez L, Major BP, Teo WP, Byrne LK, Enticott PG. The impact of stimulation intensity and coil type on reliability and tolerability of cerebellar brain inhibition (CBI) via dual-coil TMS. Cerebellum. 2018;17(5):540–9. 10.1007/s12311-018-0942-5. 29730789 10.1007/s12311-018-0942-5

[43] Faul F, Erdfelder E, Lang AG, Buchner A. G*power 3: a flexible statistical power analysis program for the social, behavioral, and biomedical sciences. Behav Res Methods. 2007;39(2):175–91. 10.3758/BF03193146. 17695343 10.3758/bf03193146

[44] Marchini A, Pedroso W, Neto OP. Mixed Modal Training to Help Older Adults Maintain Postural Balance. J Chiropr Med [Internet]. 2020 Sep 1 [cited 2025 Jul 20];18(3):198. Available from: https://pmc.ncbi.nlm.nih.gov/articles/PMC7452169/10.1016/j.jcm.2019.01.003 PMC7452169 32874159

[45] Li Z, Liang YY, Wang L, Sheng J, Ma SJ. Reliability and validity of center of pressure measures for balance assessment in older adults. J Phys Ther Sci [Internet]. 2016 Apr 1 [cited 2025 Jul 20];28(4):1364. Available from: https://pmc.ncbi.nlm.nih.gov/articles/PMC4868244/10.1589/jpts.28.1364 PMC4868244 27190484

[46] Granacher U, Muehlbaue T, Zahner L, Gollhofer A, Kressig RW. Comparison of traditional and recent approaches in the promotion of balance and strength in older adults. Sports Medicine [Internet]. 2011 [cited 2025 Jul 20];41(5):377–400. Available from: https://pubmed.ncbi.nlm.nih.gov/21510715/10.2165/11539920-000000000-00000 21510715

[47] Low DC, Walsh GS, Arkesteijn M. Effectiveness of exercise interventions to improve postural control in older adults: a systematic review and meta-analyses of centre of pressure measurements. Sports Med. 2017;47(1):101–12. 10.1007/s40279-016-0559-0. 27245061 10.1007/s40279-016-0559-0 PMC5215248

[48] Van Malderen S, Hehl M, Nuyts M, Verstraelen S, Heemels RE, Hardwick RM, et al. Age-related differences in task-related modulation of cerebellar brain inhibition. Neurobiol Aging. 2025;150:53–68. 40068243 10.1016/j.neurobiolaging.2025.02.009

[49] Mooney RA, Casamento-Moran A, Celnik PA. The reliability of cerebellar brain inhibition. Clin Neurophysiol. 2021;132(10):2365–70. 34454263 10.1016/j.clinph.2021.06.035 PMC11787817

[50] Fernandez L, Major BP, Teo WP, Byrne LK, Enticott PG. Assessing cerebellar brain inhibition (CBI) via transcranial magnetic stimulation (TMS): A systematic review. Neurosci Biobehav Rev [Internet]. 2018 Mar 1 [cited 2025 Jul 21];86:176–206. Available from: https://www.sciencedirect.com/science/article/abs/pii/S014976341730698X?via%3Dihub 10.1016/j.neubiorev.2017.11.018 29208533

[51] Torriero S, Oliveri M, Koch G, Lo Gerfo E, Salerno S, Ferlazzo F, et al. Changes in cerebello-motor connectivity during procedural learning by actual execution and observation. J Cogn Neurosci. 2011;23(2):338–48. 10.1162/jocn.2010.21471. 20350172 10.1162/jocn.2010.21471

[52] Baarbé J, Yielder P, Daligadu J, Behbahani H, Haavik H, Murphy B. A novel protocol to investigate motor training-induced plasticity and sensorimotor integration in the cerebellum and motor cortex. J Neurophysiol. 2014;111(4):715–21. 10.1152/jn.00661.2013. 24259550 10.1152/jn.00661.2013

[53] Jayaram G, Galea JM, Bastian AJ, Celnik P. Human Locomotor Adaptive Learning Is Proportional to Depression of Cerebellar Excitability. Cerebral Cortex August [Internet]. 2011;21:1901–9. Available from: https://academic.oup.com/cercor/article/21/8/1901/269000 10.1093/cercor/bhq263 PMC3202738 21239392

[54] Schlerf JE, Galea JM, Bastian AJ, Celnik PA. Dynamic Modulation of Cerebellar Excitability for Abrupt, But Not Gradual, Visuomotor Adaptation. Journal of Neuroscience [Internet]. 2012 Aug 22 [cited 2025 Mar 31];32(34):11610–7. Available from: https://www.jneurosci.org/content/32/34/11610 10.1523/JNEUROSCI.1609-12.2012 PMC3435878 22915105

[55] Spampinato DA, Block HJ, Celnik PA. Cerebellar–M1 Connectivity Changes Associated with Motor Learning Are Somatotopic Specific. Journal of Neuroscience [Internet]. 2017 Mar 1 [cited 2025 Mar 31];37(9):2377–86. Available from: https://www.jneurosci.org/content/37/9/2377 10.1523/JNEUROSCI.2511-16.2017 PMC5354349 28137969

[56] Dijkstra BW, Bekkers EMJ, Gilat M, de Rond V, Hardwick RM, Nieuwboer A. Functional neuroimaging of human postural control: a systematic review with meta-analysis. Neurosci Biobehav Rev. 2020;115:351–62. 32407735 10.1016/j.neubiorev.2020.04.028

[57] Morton SM, Bastian AJ. Cerebellar Control of Balance and Locomotion. The Neuroscientist [Internet]. 2004 Jun [cited 2025 Mar 31];10(3):247–59. Available from: https://journals.sagepub.com/doi/10.1177/1073858404263517 10.1177/1073858404263517 15155063

[58] Surgent OJ, Dadalko OI, Pickett KA, Travers BG. Balance and the brain: a review of structural brain correlates of postural balance and balance training in humans. Gait Posture. 2019;71:245–52. 31082657 10.1016/j.gaitpost.2019.05.011 PMC6594858

[59] Stoodley CJ. The cerebellum and cognition: evidence from functional imaging studies. Cerebellum. 2012;11(2):352–65. 10.1007/s12311-011-0260-7. 21373864 10.1007/s12311-011-0260-7

[60] Schmahmann JD. The cerebellum and cognition. Neurosci Lett. 2019;688:62–75. 29997061 10.1016/j.neulet.2018.07.005

[61] King M, Hernandez-Castillo CR, Poldrack RA, Ivry RB, Diedrichsen J. Functional boundaries in the human cerebellum revealed by a multi-domain task battery. Nat Neurosci [Internet]. 2019 Aug 1 [cited 2024 Sep 24];22(8):1371–9. Available from: https://go.gale.com/ps/i.do?p=HRCA%26;sw=w%26;issn=10976256%26;v=2.1%26;it=r%26;id=GALE%7CA594583448%26;sid=googleScholar%26;linkaccess=fulltext 10.1038/s41593-019-0436-x PMC8312478 31285616

[62] Stoodley CJ, Schmahmann JD. Functional topography in the human cerebellum: a meta-analysis of neuroimaging studies. Neuroimage. 2009;44(2):489–501. 18835452 10.1016/j.neuroimage.2008.08.039

[63] Keren Happuch E, Chen SHA, Ho MHR, Desmond JE. A meta-analysis of cerebellar contributions to higher cognition from PET and fMRI studies. Hum Brain Mapp [Internet]. 2012 Feb [cited 2025 Mar 31];35(2):593. Available from: https://pmc.ncbi.nlm.nih.gov/articles/PMC3866223/10.1002/hbm.22194 PMC3866223 23125108

[64] Jacobi H, Faber J, Timmann D, Klockgether T. Update cerebellum and cognition. J Neurol. 2021;268(10):3921–5. 10.1007/s00415-021-10486-w. 33656586 10.1007/s00415-021-10486-w PMC8463403

[65] Riedel MC, Ray KL, Dick AS, Sutherland MT, Hernandez Z, Fox PM, et al. Meta-analytic connectivity and behavioral parcellation of the human cerebellum. Neuroimage. 2015;117:327–42. 25998956 10.1016/j.neuroimage.2015.05.008 PMC4512917

[66] O’Reilly JX, Beckmann CF, Tomassini V, Ramnani N, Johansen-Berg H. Distinct and Overlapping Functional Zones in the Cerebellum Defined by Resting State Functional Connectivity. Cerebral Cortex [Internet]. 2010 Apr 1 [cited 2025 Mar 31];20(4):953–65. Available from: 10.1093/cercor/bhp157 10.1093/cercor/bhp157 PMC2837094 19684249

[67] Habas C, Kamdar N, Nguyen D, Prater K, Beckmann CF, Menon V et al. Distinct Cerebellar Contributions to Intrinsic Connectivity Networks. Journal of Neuroscience [Internet]. 2009 Jul 1 [cited 2024 Apr 28];29(26):8586–94. Available from: https://www.jneurosci.org/content/29/26/8586 10.1523/JNEUROSCI.1868-09.2009 PMC2742620 19571149

[68] Krienen FM, Buckner RL. Segregated fronto-cerebellar circuits revealed by intrinsic functional connectivity. Cereb Cortex. 2009;19(10):2485–97. 10.1093/cercor/bhp135. 19592571 10.1093/cercor/bhp135 PMC2742600

[69] Hardwick RM, Therrien AS, Lesage E. Non-invasive stimulation of the motor cerebellum has potential cognitive confounds. Brain Stimul [Internet]. 2021 Jul 1 [cited 2025 Feb 24];14(4):922–3. Available from: https://www.brainstimjrnl.com/action/showFullText?pii=S1935861X21001169 10.1016/j.brs.2021.05.011 34089926

[70] Habas C, Cabanis EA. Anatomical parcellation of the brainstem and cerebellar white matter: a preliminary probabilistic tractography study at 3 T. Neuroradiology. 2007;49(10):849–63. 10.1007/s00234-007-0267-4. 17701168 10.1007/s00234-007-0267-4

[71] Bernard JA, Peltier SJ, Benson BL, Wiggins JL, Jaeggi SM, Buschkuehl M, et al. Dissociable functional networks of the human dentate nucleus. Cereb Cortex. 2014;24(8):2151–9. 10.1093/cercor/bht065. 23513045 10.1093/cercor/bht065 PMC4089384

[72] Stoodley CJ, Schmahmann JD. Functional Topography of the Human Cerebellum. Essentials of Cerebellum and Cerebellar Disorders: A Primer for Graduate Students [Internet]. 2016 Jan 1 [cited 2025 Aug 22];373–81. Available from: https://link.springer.com/chapter/10.1007/978-3-319-24551-5_51

[73] Popa T, Russo M, Meunier S. Long-lasting inhibition of cerebellar output. Brain Stimul [Internet]. 2010 Jul [cited 2025 Aug 23];3(3):161–9. Available from: https://pubmed.ncbi.nlm.nih.gov/20633445/10.1016/j.brs.2009.10.001 20633445

[74] Pauly MG, Steinmeier A, Bolte C, Hamami F, Tzvi E, Münchau A et al. Cerebellar rTMS and PAS effectively induce cerebellar plasticity. Scientific Reports 2021 11:1 [Internet]. 2021 Feb 4 [cited 2025 Aug 23];11(1):1–13. Available from: https://www.nature.com/articles/s41598-021-82496-7 10.1038/s41598-021-82496-7 PMC7862239 33542291

[75] Loo CK, Taylor JL, Gandevia SC, McDarmont BN, Mitchell PB, Sachdev PS. Transcranial magnetic stimulation (TMS) in controlled treatment studies: are some sham forms active? Biol Psychiatry [Internet]. 2000 Feb 15 [cited 2025 Aug 22];47(4):325–31. Available from: https://www.sciencedirect.com/science/article/pii/S0006322399002851?casa_token=wKfzFKZS43gAAAAA:gp6_y5FXDRobwxdr51DBk6cZe_UmKceZM8nfTZlN10ADvr8hWT75MDcDBUh6F07lq8A1hcEbIRY 10.1016/s0006-3223(99)00285-1 10686267

