# Supplementary material for: Enhancing Balance Control in Aging Through Cerebellar Theta-Burst Stimulation
Source: Cerebellum. 2025 Oct 8;24(6):161. doi: 10.1007/s12311-025-01915-x (PMC12507971; doi:10.1007/s12311-025-01915-x)

**Supplementary Figure 1:** Box and whisker plotsl with scattered dots indicated each subject, for 95% COP area for Active (Panel A) and Sham (Panel B) groups, and for CBI for Active (Panel C) and Sham (Panel D) groups. (Statistical analysis i.e. ANOVA results do not differ when the single participant who appears as an outlier in panel B, particularly at timepoint POST1, is included vs excluded. Hence, all statistical analysis results reported in the main manuscript include all participants.)


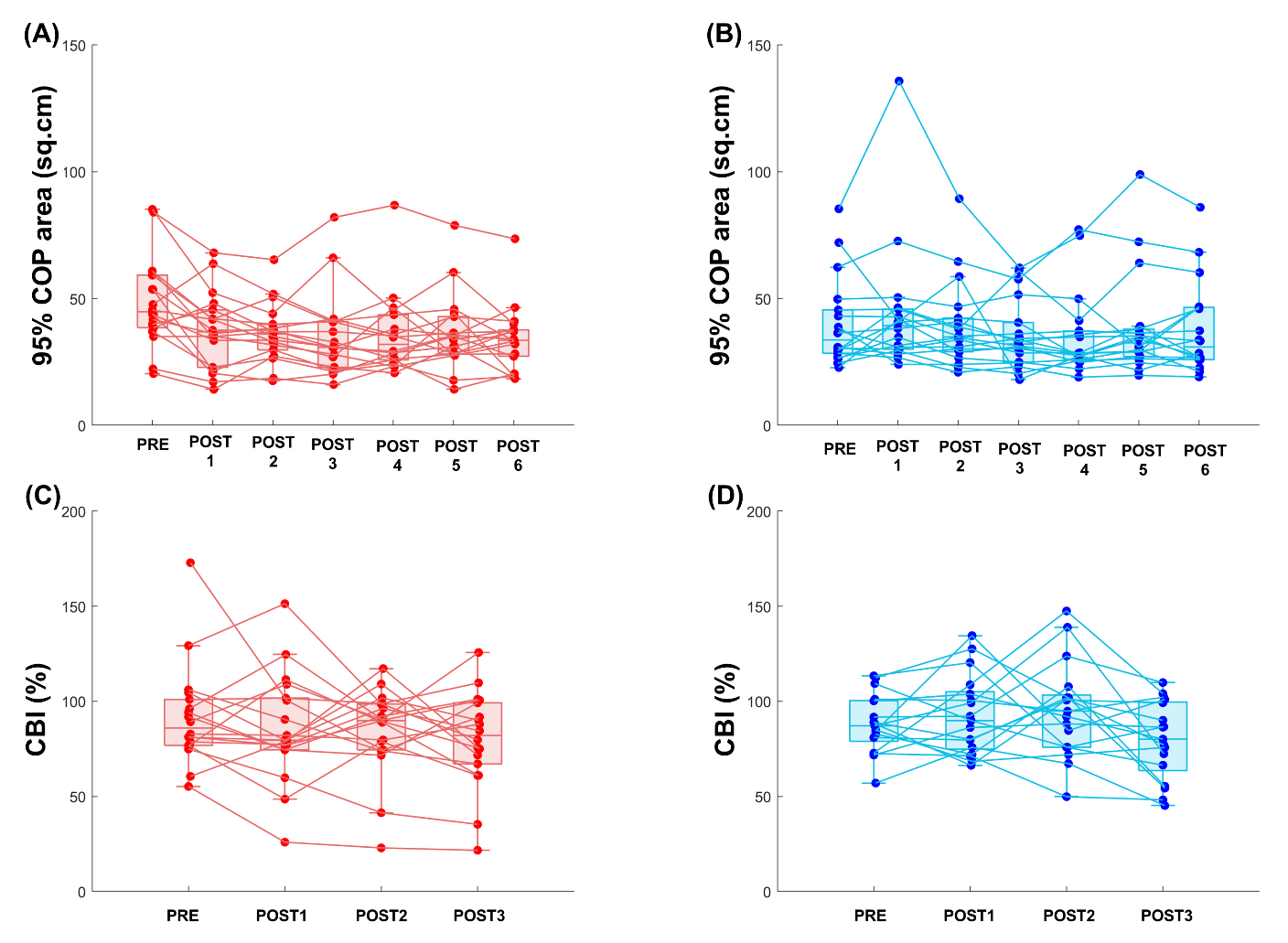

Supplement: Supplementary file 1 — Supplementary Material 1 (DOCX 463 KB) [file 12311_2025_1915_MOESM1_ESM.docx]
